# DDH-Net: A Graf-Compliant Method for Computer-Aided Assessment Using Infant Hip Ultrasound

**DOI:** 10.3390/bioengineering13091079

**Published:** 2026-09-17

**Authors:** Xinyu Zhang, Jianwei Cui, Yuxiang Dai, Wenyi Zhang

**Affiliations:** Institute of Instrument Science and Engineering, Southeast University, Nanjing 210096, China; 220253715@seu.edu.cn (X.Z.); 220243737@seu.edu.cn (Y.D.); 220243765@seu.edu.cn (W.Z.)

**Keywords:** developmental dysplasia of the hip, infant hip ultrasonography, Graf method, standard plane detection, anatomical structure segmentation, automated angle measurement

## Abstract

Ultrasound assessment of developmental dysplasia of the hip (DDH) in infants is highly operator-dependent, particularly during standard plane acquisition and Graf angle measurement. This paper proposes DDH-Net, a fully automated computer-aided assessment method for infant hip ultrasound that follows the complete Graf workflow. First, YOLOv8n-pose is used to detect eight anatomical structures and two ilium orientation keypoints for standard plane determination. Regions of interest (ROIs) are then cropped according to the detection results, and U-Net is employed to perform fine segmentation of the ilium, labrum, and lower limb of the ilium. Finally, Graf reference lines are constructed from the segmentation results to enable automatic measurement of the α and β angles. The study included 1409 infants, comprising 2648 ultrasound images and 50 ultrasound videos. On an independent test set of 529 images, the accuracy, sensitivity, and specificity of standard plane detection were 96.8%, 94.5%, and 100.0%, respectively. The model automatically saved 187 candidate frames from the videos, of which 93.1% were rated by experts as having no or only minor clinically relevant discrepancies; 48 of the 50 videos (96.0%) contained at least one clinically acceptable candidate frame. Compared with full-image segmentation, ROI-based segmentation reduced processing time by 33.7%. In 307 standard plane images, the mean absolute errors (MAE) for the α and β angles were 1.61° and 2.13°, respectively, with corresponding intraclass correlation coefficients (ICC) of 0.913 and 0.766. The complete pipeline achieved a processing speed of 28.87 frames per second, indicating that DDH-Net has the potential to provide efficient and interpretable analysis of infant hip ultrasound images.

## 1. Introduction

Developmental dysplasia of the hip (DDH) is one of the most common orthopedic disorders in neonates. It refers to an abnormal relationship between the femoral head and the acetabulum and encompasses abnormalities in the structure and morphology of osseous, cartilaginous, and soft-tissue components [1]. Epidemiological data indicate substantial geographic and ethnic variation in the incidence of DDH, ranging from 0.06‰ in African populations to 7.61% among Native American populations [2]. Early diagnosis of DDH is generally associated with favorable treatment outcomes, whereas delayed diagnosis and treatment may result in abnormal hip development, early-onset hip osteoarthritis in adulthood, and a reduced quality of life [3]. Therefore, early and accurate diagnosis of DDH is essential.

**Figure 1:**
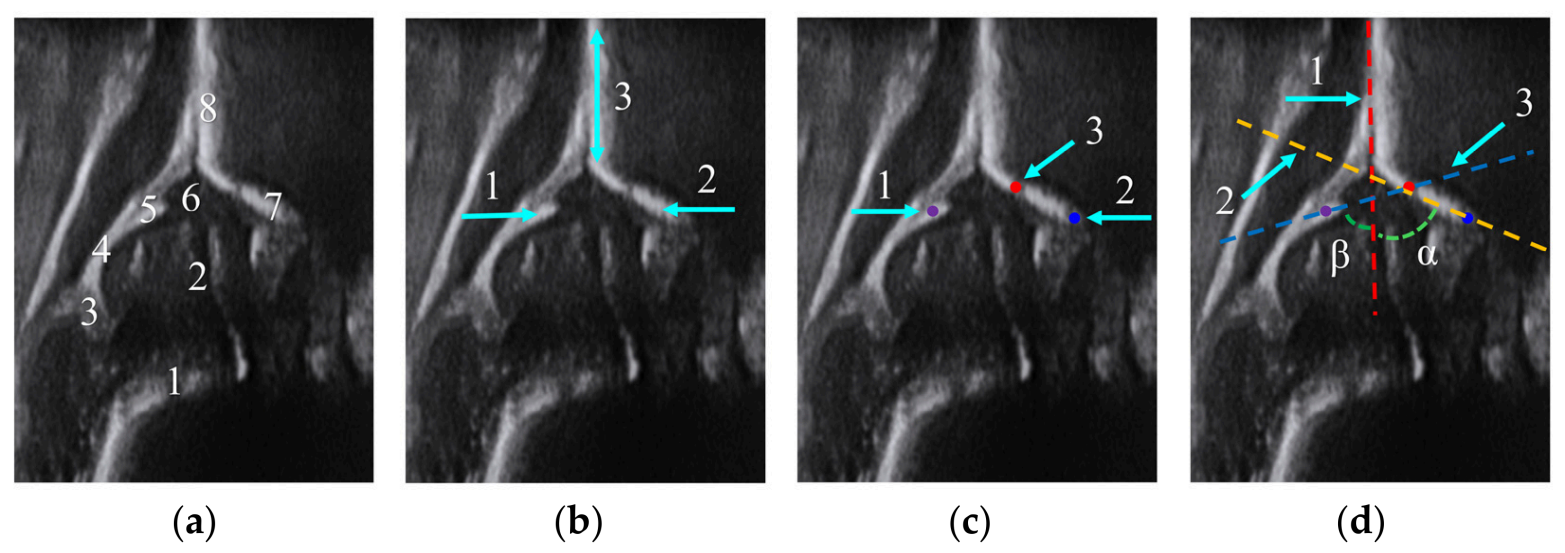
Schematic illustration of the Graf method. (**a**) Standard plane ultrasound image showing eight anatomical structures: 1, chondro-osseous border (ChB); 2, femoral head; 3, synovial fold; 4, joint capsule; 5, labrum; 6, cartilage; 7, lower limb of the ilium; and 8, middle portion of the acetabular roof. (**b**) Three key anatomical structures: 1, labrum; 2, lower limb of the ilium; and 3, ilium. (**c**) Three measurement landmarks: 1, labrum center; 2, lower limb point; and 3, bony rim point. (**d**) Three measurement lines: 1, the baseline, defined as the distally directed tangent along the echogenic contour of the ilium; 2, the bony roof line, defined as the tangent drawn from the lower-limb point along the inferior contour of the ilium; and 3, the cartilage roof line, defined as the line connecting the center of the labrum and the bony rim point. The α angle is the angle between the baseline and the bony roof line, whereas the β angle is the angle between the baseline and the cartilage roof line.

Ultrasonography provides clear visualization of the infant hip joint and surrounding soft-tissue anatomy. Owing to its lack of ionizing radiation, relatively low cost, and good repeatability, it has become the imaging modality of choice for the early screening of DDH [4]. In 1980, Graf introduced a static ultrasound examination method, which has since undergone extensive development and become a principal approach for the ultrasonographic assessment of DDH [5,6]. The Graf method is based on a standard plane (Figure 1a), on which three key anatomical structures are identified (Figure 1b). The corresponding measurement landmarks are then determined (Figure 1c), and the baseline, bony roof line, and cartilaginous roof line are constructed to calculate the α and β angles (Figure 1d) [7].

Although the Graf method is widely used in clinical practice, its diagnostic accuracy remains influenced by operator experience, ultrasound image quality, and the subjectivity of angle measurements. Previous studies have reported considerable intra- and inter-observer variability in Graf angle measurements, potentially resulting in inconsistent differentiation between normal and dysplastic hips and, in some cases, changes in severity grading and subsequent treatment decisions [8,9]. Therefore, developing an accurate, reliable, and efficient automated method for DDH diagnosis is of substantial clinical value for reducing observer dependence and improving diagnostic consistency.

Research on computer-aided diagnosis of DDH began relatively early, with methodological development progressing primarily from conventional image-processing and optimization techniques to deep-learning-based approaches.

Early studies primarily employed conventional image-processing and optimization methods to identify key anatomical structures and subsequently measure the Graf angles. Quader et al. [10] measured relevant anatomical parameters based on the acoustic and image characteristics of bone and cartilage tissues. They subsequently extended this approach to automatically identify bony and cartilaginous boundaries and calculate key DDH-related indices using the same features [11]. Hareendranathan et al. [12] proposed a semiautomated method for tracing bone-surface contours to measure the α angle and rounding index. Sezer et al. [13] combined particle swarm optimization with a statistical level-set method to localize the region of interest, segment key anatomical structures, and calculate the α and β angles. However, these methods generally rely on handcrafted features, initialization parameters, and prior rules, resulting in limited robustness and generalizability when applied to ultrasound images with low contrast, severe speckle noise, or incomplete visualization of anatomical structures.

With the rapid development of deep learning, convolutional neural networks (CNNs) have been increasingly applied to DDH diagnosis, with related studies primarily focusing on key anatomical structure segmentation, automated Graf angle measurement, and standard plane detection. For anatomical structure segmentation, Zhang et al. [14] and El-Hariri et al. [15] improved the segmentation accuracy of the ilium and labrum by introducing a localization unit and a multichannel feature-extraction strategy, respectively. Pulik et al. [16] systematically compared multiple semantic segmentation networks using a large-scale hip ultrasound dataset and achieved accurate identification of key anatomical structures and landmarks. For automated measurement, Golan et al. [17] were among the first to use deep learning to segment the ilium and acetabular roof and subsequently calculate the α angle. Hu et al. [18] proposed a multitask learning framework that jointly performed the detection, segmentation, and localization of key anatomical structures within a single network and generated the corresponding angle measurements. Lee et al. [19] developed a two-stage network that further predicted measurement landmarks based on the segmentation results and used them to calculate the α and β angles. Huang et al. [20] proposed a lightweight diagnostic system that integrated key-structure detection and segmentation to automatically measure multiple indices, including the α angle, β angle, femoral head coverage, and femoral head displacement distance. For standard plane detection, Liu et al. [21] proposed NHBS-Net, which determined whether an image satisfied the standard plane criteria by segmenting multiple key anatomical structures. Ahn et al. [22] achieved standard plane classification by integrating global and local features with multiple anatomical landmarks. Chen et al. [23] further developed a method for standard plane quality scoring based on the detection results for the ilium, labrum, lower limb of the os ilium, and bony rim, while simultaneously performing Graf angle measurements.

**Figure 2:**
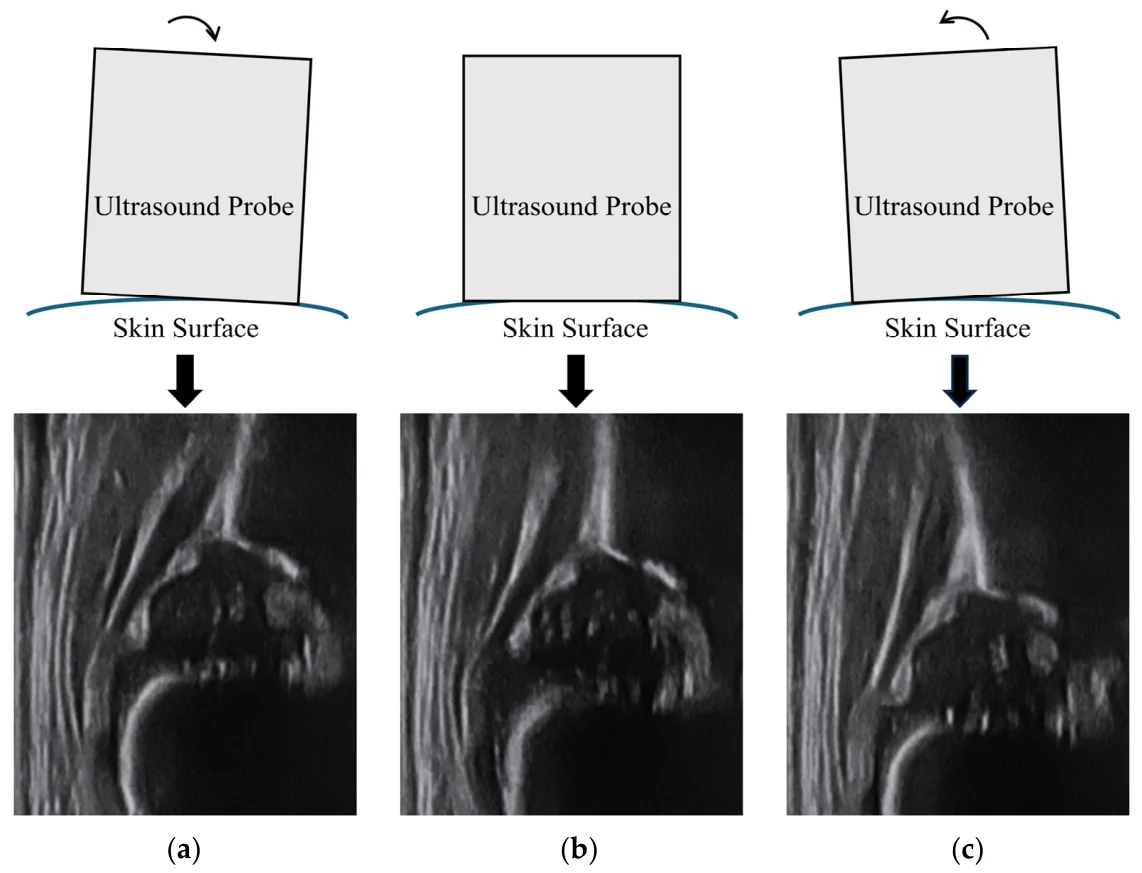
Schematic illustration of standard plane deviation caused by variations in probe posture. (**a**) Posterior section, with the iliac contour inclined to the right and extending away from the probe. (**b**) Middle cross section corresponding to the Graf standard plane, with an approximately straight iliac contour roughly parallel to the probe. (**c**) Anterior section, with the iliac contour inclined to the left and extending toward the probe. The inclined iliac contours in (**a**,**c**) indicate that the imaging plane deviates from the central portion of the acetabular roof and therefore does not satisfy the standard plane requirements of the Graf method. The curved arrows indicate the direction of probe tilting, while the downward arrows indicate the corresponding ultrasound images acquired at different probe orientations.

Although the studies described above have made substantial progress in the automated diagnosis of DDH, several limitations remain. (1) Accurate standard plane determination is a prerequisite for reliable angle measurement. However, most existing methods either assume that the input images are standard plane images or perform only a coarse assessment based on a subset of anatomical features, without effectively constraining out-of-plane tilt. In clinical examinations, changes in probe orientation may cause the imaging plane to deviate from the standard plane, as illustrated in Figure 2. Although structures such as the ilium, labrum, and lower limb of the ilium may remain visible, an inclined iliac contour indicates that the imaging plane does not accurately pass through the middle of the acetabular roof and therefore fails to satisfy the standard plane requirements of the Graf method [24]. (2) Some studies do not strictly follow the Graf diagnostic workflow but instead calculate the α and β angles directly from model-predicted measurement landmarks, omitting essential steps such as standard plane assessment and key anatomical structure identification. Consequently, the resulting measurements may lack sufficient clinical interpretability and reliability. (3) Existing studies generally focus on an individual task, such as standard plane determination, anatomical landmark segmentation, measurement landmark localization, or angle calculation. A complete framework that integrates these components into the full Graf diagnostic workflow has yet to be established, limiting the realization of fully automated analysis of infant hip ultrasound images.

To address these limitations, this study proposes DDH-Net, a fully automated computer-aided assessment method for infant hip ultrasonography that integrates deep learning with geometric measurement. Following the diagnostic workflow of the Graf method, DDH-Net sequentially performs standard plane detection, segmentation of key anatomical structures, and automated measurement of the α and β angles. First, DDH-Net determines whether the input image satisfies the Graf standard plane criteria by jointly evaluating the completeness of anatomical structures and the orientation of the ilium. Subsequently, regions of interest are extracted according to the detection results, followed by fine segmentation of the ilium, labrum, and lower limb of the ilium. Finally, measurement landmarks are identified from the segmented contours, and the baseline, bony roof line, and cartilage roof line are automatically constructed to calculate the Graf angles. In addition, the standard plane detection module can perform frame-by-frame analysis of continuous ultrasound videos and automatically save candidate standard plane images that meet the predefined criteria. The main contributions of this study are as follows:

(1) A fully automated computer-aided assessment method, termed DDH-Net, is proposed for infant hip ultrasonography. By following the Graf diagnostic workflow, the method integrates standard plane detection, key anatomical structure segmentation, and Graf angle measurement, thereby improving ultrasound image analysis efficiency and the interpretability of the measurement process.
(2) A standard plane detection module integrating anatomical structure detection and ilium orientation assessment is designed. Standard plane quality control is achieved by jointly evaluating anatomical structure completeness and the degree of iliac inclination. The method also supports frame-by-frame analysis of continuous ultrasound videos and the automatic saving of candidate standard plane images.
(3) An object-detection-guided method for region-of-interest (ROI) segmentation and geometric measurement is proposed. Detection-guided ROI extraction and U-Net segmentation reduce background interference and computational overhead. The resulting segmentation contours are then used to construct the Graf reference lines and automatically calculate the α and β angles, thereby improving the segmentation of small anatomical structures and the interpretability of the measurement process.

## 2. Materials and Methods

This section first describes the construction, annotation, and partitioning of the dataset, followed by an overview of the proposed DDH-Net architecture and its functional modules to provide a comprehensive understanding of the overall workflow. The model training procedure and parameter settings are then presented. Finally, the performance evaluation metrics and statistical analysis methods used in this study are introduced.

### 2.1. Dataset and Annotation

Between June and December 2025, a total of 1409 infants aged 0–6 months who underwent hip ultrasonography at Yangzhou Maternal and Child Health Care Hospital were included in this study. The mean age was 55.9 ± 26.8 days (range, 28–163 days), and the cohort comprised 573 male infants (40.67%) and 836 female infants (59.33%). The eligibility of the ultrasound images was assessed and confirmed by experienced clinicians according to the predefined inclusion and exclusion criteria. Following data screening, a total of 2648 hip ultrasound images and 50 ultrasound video clips were included. Each infant contributed one or two images, representing one or both hips, and only one image per hip was retained in the final dataset. All ultrasound images were acquired using a D8C handheld ultrasound imaging system, anonymized, and stored in Digital Imaging and Communications in Medicine format.

Each ultrasound image was first assessed by an expert with more than 20 years of clinical experience to determine whether it represented a Graf standard plane. A standard plane image was required to meet the following two criteria according to the Graf method [5]: (1) all eight anatomical structures were clearly visible; and (2) the ilium was vertically oriented. To quantitatively characterize the latter criterion for automated assessment, an absolute ilium inclination angle of less than 5° was adopted in this study based on visual assessment of the ultrasound images in consultation with experienced clinical experts. Following this screening process, 1532 images that satisfied the Graf criteria for a standard plane and 1116 images classified as nonstandard planes were obtained.

**Figure 3:**
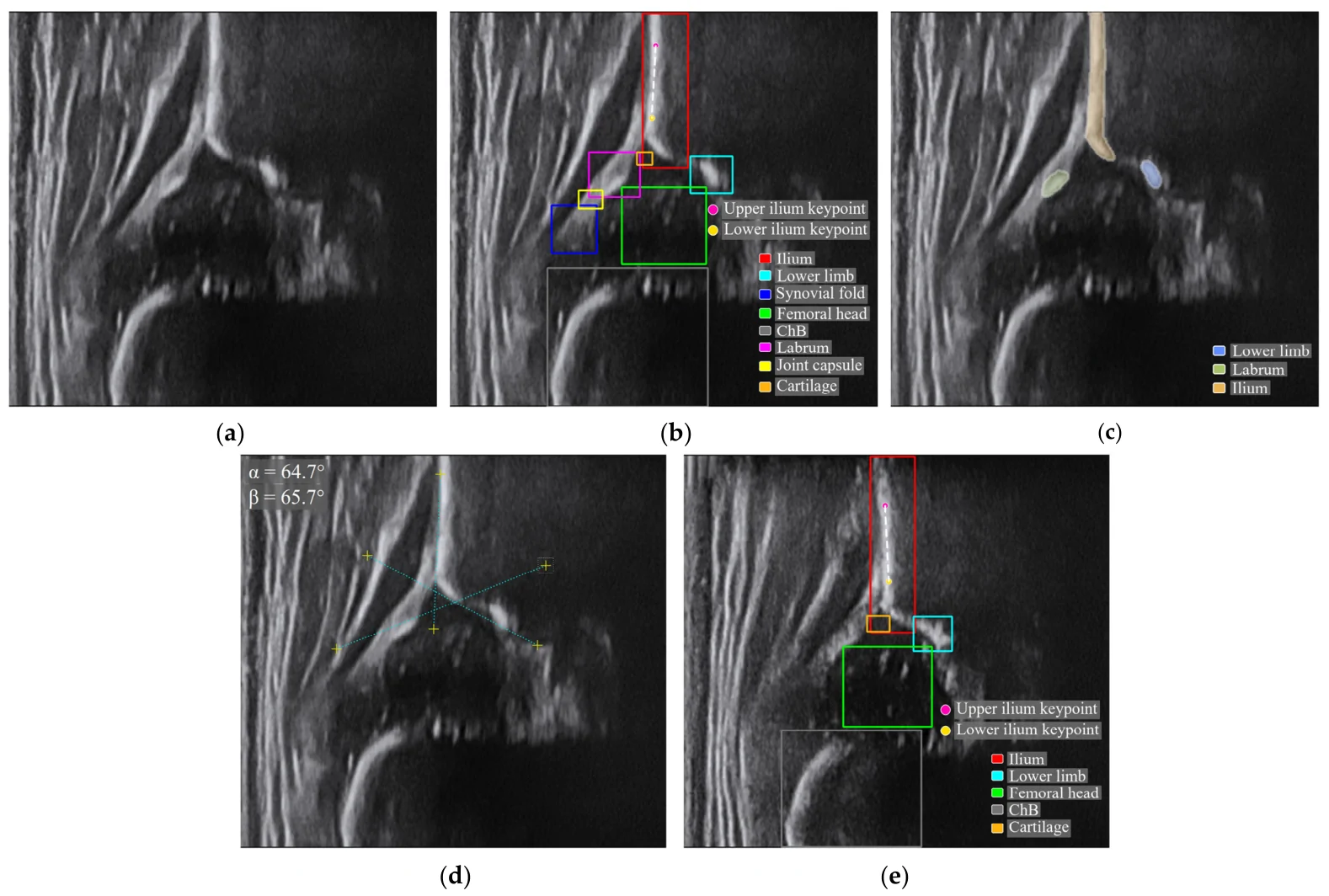
Annotation protocol for the infant hip ultrasound dataset. (**a**) Original Graf standard plane ultrasound image. (**b**) Bounding-box annotations of eight anatomical structures and two ilium keypoints. (**c**) Manual segmentation masks of the ilium, labrum, and lower limb of the ilium. (**d**) Manual measurement of the Graf α and β angles; the dashed lines represent the measurement lines, and the symbols indicate the endpoints used to draw these lines. (**e**) Annotation example of a nonstandard plane image, in which only clearly visible anatomical structures and identifiable ilium keypoints were annotated.

Subsequently, task-specific annotation datasets were constructed, with representative annotation examples shown in Figure 3. For standard plane images, bounding-box annotations were provided for the eight visible anatomical structures, and two keypoints were annotated on the ilium to characterize its orientation. Meanwhile, the three key anatomical structures—the ilium, labrum, and lower limb of the ilium—were manually segmented, and reference measurements of the α and β angles were obtained according to the Graf method. For each nonstandard plane image, bounding-box annotations were provided only for the anatomical structures that were clearly visible in the image. When the ilium could be reliably identified, the two keypoints describing its orientation were also annotated. Nonstandard plane images were excluded from manual Graf angle measurement and U-Net segmentation training and were used primarily to improve the model’s ability to recognize nonstandard planes and images with incomplete visualization of the relevant anatomical structures.

To ensure the reliability of the annotations and measurements, all standard plane classifications, bounding-box annotations, keypoint annotations, segmentation masks, and Graf angle measurements initially obtained by the primary expert were independently reviewed by two additional experts with extensive clinical experience. When discrepancies were identified, the corresponding annotations, measurement landmarks, reference lines, and angle measurements were reassessed, and the final reference measurements were established through consensus among the three experts. The dataset was partitioned at the infant level to prevent information leakage between subsets. The 1409 infants were randomly assigned to the training, validation, and test subsets at an approximate ratio of 7:1:2. All ultrasound images obtained from the same infant, including bilateral hip images when available, were assigned exclusively to the same subset. The resulting training, validation, and test sets were used for YOLOv8-pose model training, hyperparameter optimization, and final performance evaluation, respectively. The standard plane subset was further used for U-Net segmentation model training and validation of the automated Graf angle measurements. The dataset allocation is summarized in Table 1. Based on the expert-measured α and β angles, the standard plane images were further classified according to the Graf classification system. Among them, 1113 images were classified as Graf type I and 419 as Graf type II. The distributions of the two Graf types across the training, validation, and test sets are presented in Table 2. The 50 ultrasound videos were obtained from 50 unique infants and were acquired during routine examinations by one experienced clinician. Each video was selected to ensure that the continuous scanning sequence passed through at least one Graf standard plane, which was confirmed by the acquiring clinician and independently reviewed by two additional experienced clinicians. The videos were not used for model training and were used exclusively for video-based evaluation.

This was a single-center retrospective study. All ultrasound images and videos were obtained from prior routine clinical examinations, and no additional examinations or interventions were performed for research purposes. The study was reviewed and approved by the Ethics Committee of Yangzhou Maternal and Child Health Care Hospital (Ethics Approval No. 021, 2024; approval date: 24 May 2024). Written informed consent was obtained from the parents or legal guardians of all infants included in the study. All data were anonymized before being used for research.

### 2.2. DDH-Net Architecture

**Figure 4:**
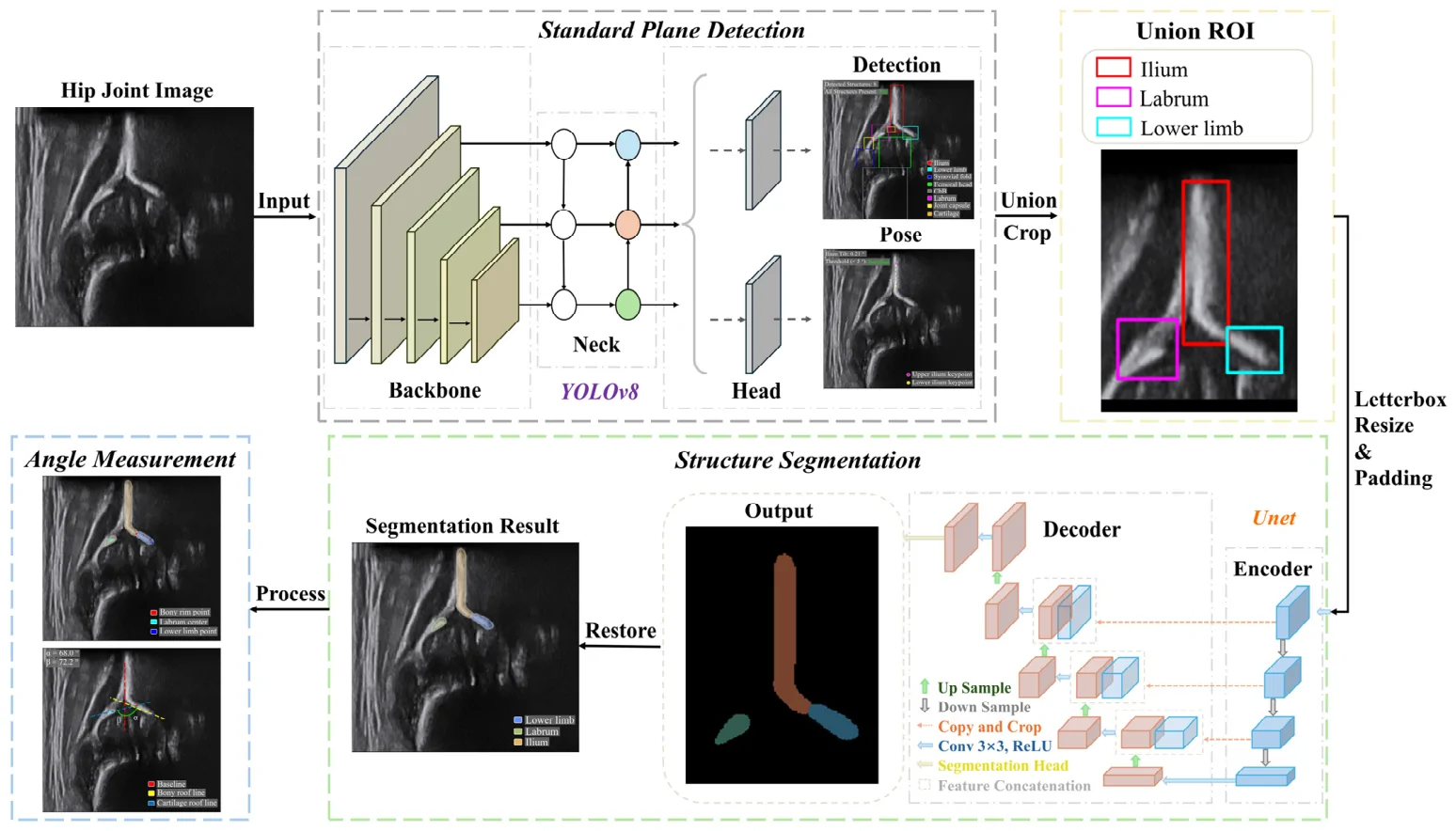
Overall architecture of DDH-Net. Arrows and connecting lines indicate the data-processing flow and network connections, colored squares and regions denote the corresponding structures or components as specified in the figure legends, and dashed boxes delineate the main functional modules.

The overall architecture of the proposed DDH-Net is illustrated in Figure 4 and consists of three main modules: standard plane detection, key anatomical structure segmentation, and Graf angle measurement. For an input infant hip ultrasound image, the standard plane detection module first determines whether the image satisfies the standard plane criteria of the Graf method. For images that meet these criteria, the regions of interest associated with Graf angle measurement are cropped according to the detection results and subsequently subjected to fine-grained segmentation using the anatomical structure segmentation module. Finally, the measurement landmarks required by the Graf method are identified from the segmentation results, and the corresponding reference lines are constructed to enable automated measurement of the α and β angles.

Based on the overall workflow described above, the implementation of each module is detailed in the following sections.

#### 2.2.1. Standard Plane Detection

Standard plane detection was primarily implemented using the YOLOv8-pose network. YOLOv8 [25] is a one-stage object detection framework comprising three main components: a backbone, a neck, and a head. The backbone extracts multiscale features, the neck integrates features at different scales through a feature pyramid structure, and the head predicts object locations and categories. Building on this architecture, YOLOv8-pose incorporates an additional keypoint regression branch into the detection head, enabling the simultaneous prediction of object bounding boxes and keypoint coordinates in a single forward pass and thereby jointly performing object detection and keypoint localization.

In this study, the YOLOv8-pose model was used to simultaneously detect eight anatomical structures and two ilium orientation keypoints. The eight anatomical structures included the ilium, lower limb of the ilium, labrum, femoral head, synovial fold, joint capsule, cartilage, and chondro-osseous border (ChB). The two orientation keypoints were annotated on the iliac cortex to characterize the direction of the iliac axis.

Based on the model outputs, the presence of all eight anatomical structures was first assessed to evaluate the completeness of anatomical visualization. Subsequently, the direction of the ilium was calculated from the two detected orientation keypoints, and its angle relative to the vertical direction was determined and defined as the ilium inclination angle. Let $P_{1}(x_{1},\;y_{1})$ and $P_{2}(x_{2},\;y_{2})$ denote the coordinates of the two detected keypoints, respectively. The direction vector of the iliac axis can then be expressed as:

(1) $$\vec{v}=(x_{2}-x_{1},\;y_{2}-y_{1})$$

The ilium inclination angle, θ, can be expressed as:

(2) $$\theta =arctan\left(\frac{x_{2}-x_{1}}{y_{2}-y_{1}}\right)$$

When all eight anatomical structures are detected and the ilium inclination angle satisfies $\left|\theta \right|<5{}^{\circ},$ the image is classified as a standard plane that meets the requirements of the Graf method; otherwise, it is considered not to satisfy the standard plane criteria. By combining the detection of anatomical structures with geometric constraints on ilium orientation, the proposed method enables automated identification of standard planes in hip ultrasound images, thereby providing a reliable basis for subsequent anatomical landmark segmentation and Graf angle measurement.

#### 2.2.2. Structure Segmentation

Following standard plane detection, the key anatomical structures were finely segmented to obtain accurate morphological information. U-Net [26] is a classical semantic segmentation network with an encoder–decoder architecture. The encoder extracts high-level semantic features through successive downsampling, whereas the decoder progressively restores spatial resolution through upsampling. Meanwhile, skip connections fuse low-level spatial details with high-level semantic information, thereby enabling accurate segmentation of small anatomical structures in ultrasound images. To improve segmentation performance in complex ultrasound images, ResNet50 was adopted as the backbone of U-Net, leveraging its strong feature extraction capability and the advantages of residual connections in facilitating the optimization of deep neural networks.

Moreover, numerous studies have demonstrated that localizing and cropping the region of interest (ROI) before fine-grained segmentation can effectively reduce interference from background tissues and improve the segmentation accuracy of small anatomical structures [27,28,29]. Based on the detection results produced by the YOLOv8-pose model, the bounding boxes corresponding to the ilium, labrum, and lower limb of the ilium were selected, and the minimum enclosing rectangle covering all three bounding boxes was calculated. To preserve the necessary contextual tissue information surrounding the target structures, the combined region was expanded by 10% along each boundary to obtain the final ROI. The ROI was then cropped from the original ultrasound image and resized to 192 × 256 pixels using aspect-ratio-preserving letterbox resizing and padding. Finally, the processed ROI was fed into the U-Net network to achieve fine-grained semantic segmentation of the three key anatomical structures.

#### 2.2.3. Graf Angle Measurement

**Figure 5:**
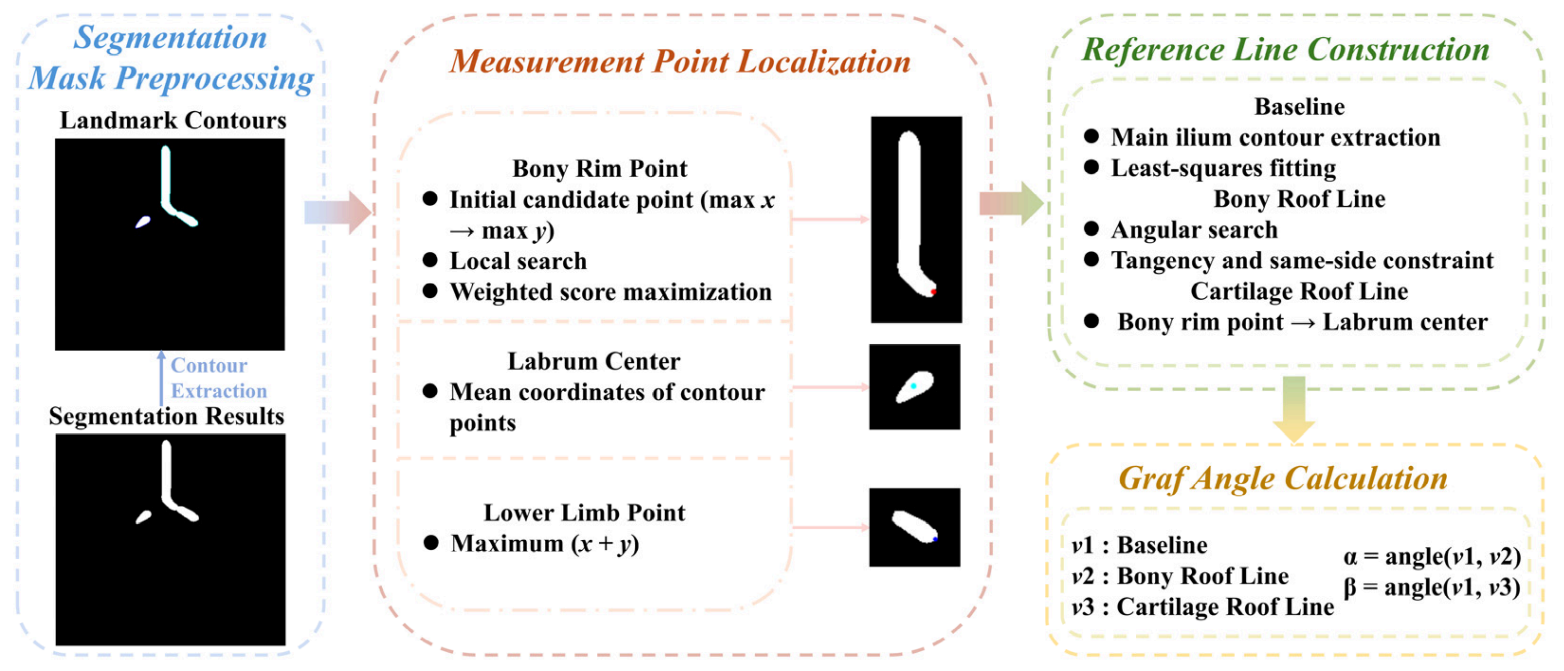
Pipeline of automatic Graf angle measurement. Arrows indicate the processing flow, while the colored dashed boxes distinguish the main stages and functional modules of the measurement pipeline.

To enable automated Graf angle measurement in infant hip ultrasound images, the bony rim point, the center of the labrum, and the lower limb point were further extracted from the anatomical structure segmentation results. Based on these measurement landmarks, the reference lines required by the Graf method were constructed, allowing the α and β angles to be calculated automatically. The complete workflow for automated Graf angle measurement is illustrated in Figure 5.

First, the segmentation masks of the ilium, labrum, and lower limb of the ilium were binarized, and the outer contour of the largest connected component in each mask was extracted. The three measurement landmarks were then localized based on the resulting contour information.

The bony rim point was defined as the lateral endpoint of the bony acetabular contour. To obtain an initial estimate, the contour point with the maximum x-coordinate was selected. If multiple points had the same maximum x-coordinate, the point with the largest y-coordinate was chosen. A local search was then performed within the neighborhood of this initial point using the following weighted scoring function:

(3) $$J=\lambda_{1}x+\lambda_{2}y$$

Here, x and y denote the horizontal and vertical coordinates of a contour point, respectively, and $\lambda_{1}$ and $\lambda_{2}$ are weighting parameters. The values of $\lambda_{1}=0.7\;$and $\lambda_{2}=1.3$ were empirically selected based on the geometric characteristics of the bony rim and visual assessment of localization performance on representative ultrasound images, with a larger weight assigned to the vertical coordinate to better reflect its expected anatomical position. The candidate point was iteratively updated by maximizing the scoring function until the search converged, yielding the final bony rim point. Given the relatively compact morphology of the labrum, its location was determined directly by calculating the geometric centroid of the labral contour points. The lower limb point corresponds to the lower-right extremity of the contour and was identified as the contour point with the maximum sum of its x- and y-coordinates.

After the three landmarks and the iliac contour had been identified, the reference lines required for Graf angle measurement were constructed. The baseline was derived from the principal contour points along the left side of the ilium. To minimize the influence of noise at the upper and lower ends of the contour, only the central portion of the iliac shaft was retained. A straight line was then fitted to the left-side contour points using the least-squares method to obtain the baseline equation. The bony roof line was determined jointly from the lower limb point and the iliac contour. Specifically, taking the lower-limb point as the pivot, lines passing through this point were evaluated over a range of orientations, and the distances from the contour points to each candidate line were calculated. The first line that became tangent to the iliac contour while all contour points remained on the same side of the line was defined as the bony roof line. Finally, the cartilage roof line was constructed by connecting the bony rim point to the center of the labrum.

After the three reference lines had been constructed, the α and β angles were calculated. Let $v_{1}$, $v_{2}$, and $v_{3}$ denote the direction vectors of the baseline, bony roof line, and cartilage roof line, respectively. The α and β angles were then calculated as follows:

(4) $$\alpha =arccos\left(\frac{v_{1}\cdot v_{2}}{\left|v_{1}\right|\cdot \left|v_{2}\right|}\right)$$

(5) $$\beta =arccos\left(\frac{v_{1}\cdot v_{3}}{\left|v_{1}\right|\cdot \left|v_{3}\right|}\right)$$

### 2.3. Network Training

The standard plane detection module was developed by fine-tuning a pretrained YOLOv8n-pose model to detect the eight anatomical structures and localize the two ilium orientation keypoints. All input images were resized to 416 × 416 pixels. The model was trained for 200 epochs with a batch size of 16 using the stochastic gradient descent optimizer and an initial learning rate of 0.01. Data augmentation techniques, including brightness adjustment, small-angle rotation, translation, and scaling, were applied during training. The model achieving the best performance on the validation set was selected for the subsequent experiments. During inference, the confidence threshold was set to 0.45, and the intersection-over-union (IoU) threshold for non-maximum suppression was set to 0.70.

The anatomical structure segmentation module was trained using a two-stage strategy. In the first stage, the minimum enclosing rectangle covering the manually annotated bounding boxes of the ilium, labrum, and lower limb of the ilium was calculated. The resulting region was then expanded by 10% along each boundary to preserve the necessary surrounding tissue information. The 10% margin was empirically selected to provide sufficient surrounding anatomical context and accommodate minor bounding-box localization errors, while limiting the inclusion of irrelevant background tissue. The ROI was cropped from the original ultrasound image and resized to 192 × 256 pixels using aspect-ratio-preserving letterbox resizing and padding for the initial training of U-Net. The model was trained for 200 epochs with a batch size of 16 using the AdamW optimizer, a cosine annealing learning-rate schedule, and a combined loss comprising weighted cross-entropy loss and Dice loss. The model with the highest mean foreground Dice score on the validation set was selected. In the second stage, the trained YOLOv8n-pose model was used to predict the bounding boxes of the three key anatomical structures. New ROIs were then generated using the same procedure for calculating the combined region, applying a 10% margin expansion, and performing letterbox resizing and padding. U-Net was further trained on these detection-derived ROIs to improve its robustness to bounding-box localization errors and scale variations encountered during actual inference. The model achieving the highest mean foreground Dice score on the validation set was selected as the final segmentation model. During the final test evaluation, ROIs were automatically generated from the bounding boxes predicted by the trained YOLOv8n-pose model and subsequently used for U-Net segmentation and Graf angle measurement.

### 2.4. Statistical Analysis

All statistical analyses were performed using SPSS software (version 27.0; IBM Corp., Armonk, NY, USA). For standard plane detection, the assessments of clinical experts were used as the reference standard, and a confusion matrix was constructed, with standard plane images defined as positive samples and nonstandard plane images as negative samples. Based on the confusion matrix, accuracy, sensitivity, specificity, positive predictive value, negative predictive value, and F1-score were calculated to comprehensively evaluate the model’s ability to distinguish standard plane images from nonstandard plane images. Sensitivity reflected the model’s ability to correctly identify standard plane images, whereas specificity reflected its ability to correctly exclude nonstandard plane images. In addition, Cohen’s kappa coefficient was used to assess agreement between the model predictions and the expert reference standard.

The clinical acceptability of automated standard plane acquisition from ultrasound video streams was evaluated through visual assessment by clinical experts. According to the degree of discrepancy between each candidate image and the clinically accepted standard plane, the results were categorized into five levels: None, Minor, Moderate, Major, and Failure. None indicated that all required anatomical structures were clearly visualized and the ilium orientation satisfied the predefined standard plane criterion, fully meeting the Graf standard plane requirements. Minor indicated slight deviations in anatomical visualization or ilium orientation that did not affect standard plane determination or subsequent Graf angle measurement. Moderate indicated noticeable deviations that could affect standard plane determination or measurement reliability and therefore required expert review. Major indicated substantial anatomical incompleteness or marked ilium deviation that made the image unsuitable for reliable Graf angle measurement. Failure indicated that the captured candidate frame could not be accepted as a valid standard plane for subsequent analysis. The feasibility of automatically capturing and saving standard plane images during continuous scanning was evaluated by calculating the number and percentage of cases in each category. In addition to frame-level evaluation, video-level performance was assessed. A video was considered successfully processed if at least one automatically captured candidate frame was rated as None or Minor by the clinical experts. The number of candidate frames obtained from each video and the corresponding video-level success rate were calculated.

For Graf angle measurement, the expert measurements confirmed through the consensus procedure described in Section 2.1 were used as the reference standard. The mean absolute error (MAE) and root mean square error (RMSE) were calculated to quantify the errors of the automated measurements. The intraclass correlation coefficient (ICC) was calculated using a two-way mixed-effects model with absolute agreement and single measurements [ICC(A,1)] to assess agreement between the automated and expert measurements. ICC values were interpreted as indicating poor agreement when ICC < 0.50, moderate agreement when 0.50 ≤ ICC < 0.75, good agreement when 0.75 ≤ ICC ≤ 0.90, and excellent agreement when ICC > 0.90 [30]. In addition, Bland–Altman analysis was performed to further evaluate the bias between the automated and expert measurements. The mean difference and corresponding 95% limits of agreement were calculated [31] to assess the level of agreement between the two methods across the clinically relevant measurement range. Subgroup analyses were further performed according to the expert-derived Graf classification. For Graf type I and II hips, the MAE and RMSE of the automated α- and β-angle measurements were calculated separately to assess whether measurement performance remained consistent across the two Graf categories represented in the dataset.

## 3. Results

### 3.1. Diagnostic Performance of Standard Plane Detection

To evaluate the discriminative performance of the standard plane detection module, the trained YOLOv8-pose model was applied to an independent test set comprising 529 hip ultrasound images, including 307 standard plane images and 222 nonstandard plane images.

The confusion matrix comparing the model predictions with the expert reference standard is presented in Table 3. Among the 307 standard plane images confirmed by the experts, 290 were correctly identified by the model, whereas 17 were missed. All 222 nonstandard plane images were correctly classified as nonstandard planes, with no false-positive predictions. The performance metrics derived from the confusion matrix are summarized in Table 4. The model achieved an accuracy of 96.8% and a sensitivity of 94.5%, indicating that it successfully identified the vast majority of standard plane images satisfying the Graf criteria. Both specificity and positive predictive value reached 100.0%, demonstrating that, in this test set, the model reliably excluded nonstandard plane images and that all images classified by the model as standard planes were consistent with the expert assessments. The F1-score was 97.2%, and Cohen’s kappa coefficient was 0.935, further indicating a high level of agreement between the model predictions and the expert reference standard.

**Figure 6:**
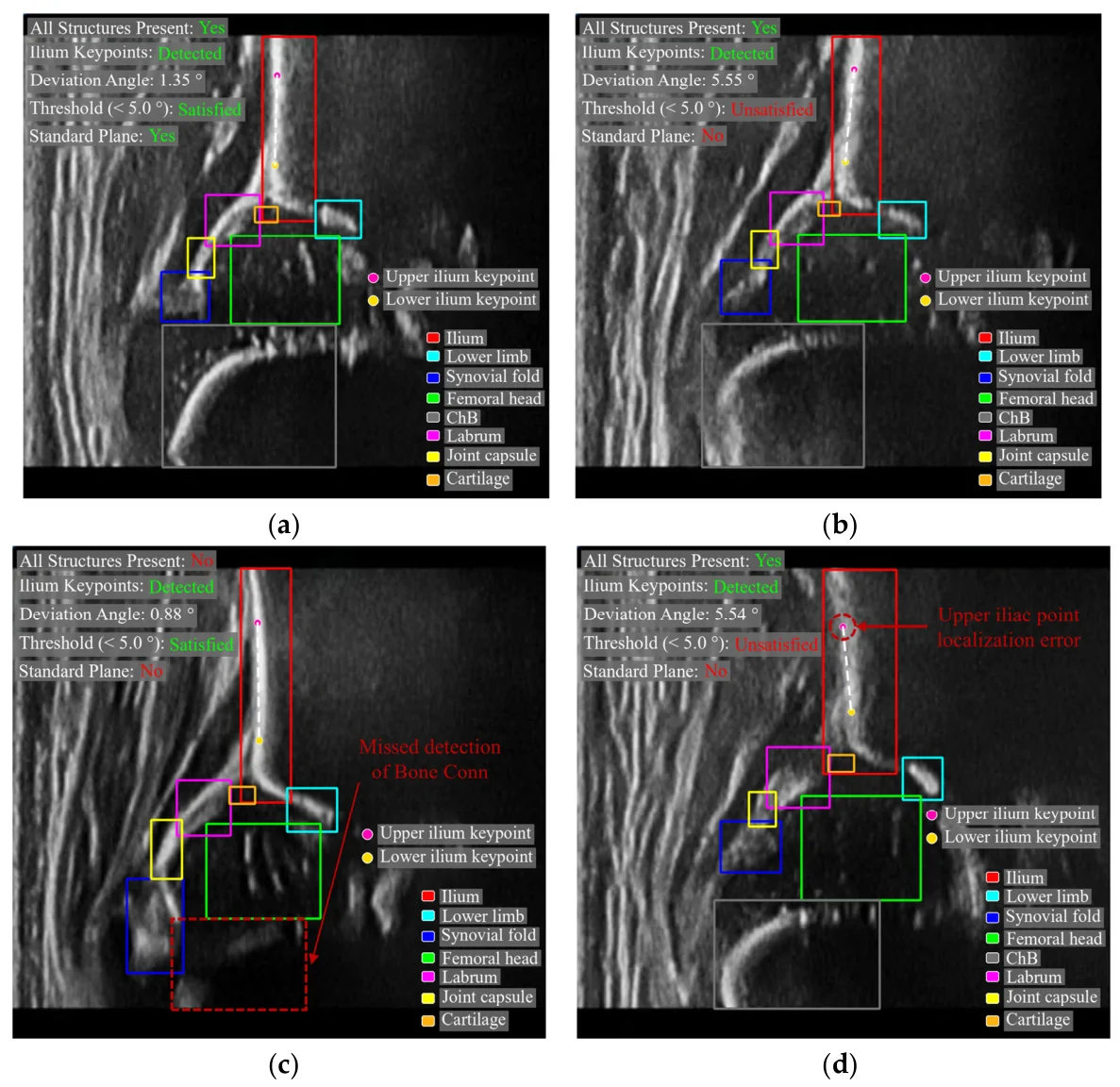
Representative examples of standard plane detection. (**a**) True positive case. (**b**) True negative case. (**c**) False-negative case caused by missed detection of the ChB. The red dashed box and arrow highlight the region associated with the missed detection. (**d**) False negative case caused by upper ilium keypoint localization error. The red dashed circle and arrow indicate the incorrectly localized upper ilium keypoint, resulting in failure to satisfy the ilium tilt criterion. No false-positive case was observed in the test set.

Figure 6 presents representative images corresponding to different classification outcomes. For standard plane images, the model successfully identified the complete set of anatomical structures and correctly classified the images by additionally considering the orientation of the ilium. Nonstandard plane images were effectively excluded. The small number of false-negative cases was primarily associated with incomplete visualization of local anatomical structures, reduced image quality, or localization errors in the ilium keypoints. No nonstandard plane image was incorrectly classified as a standard plane in the test set.

Overall, the proposed standard plane detection module accurately distinguished standard plane images from nonstandard plane images and demonstrated particularly stable performance in excluding nonstandard planes. It therefore provides reliable input for subsequent anatomical structure segmentation and automated Graf angle measurement.

### 3.2. Feasibility Evaluation of Video-Based Standard Plane Acquisition

**Figure 7:**
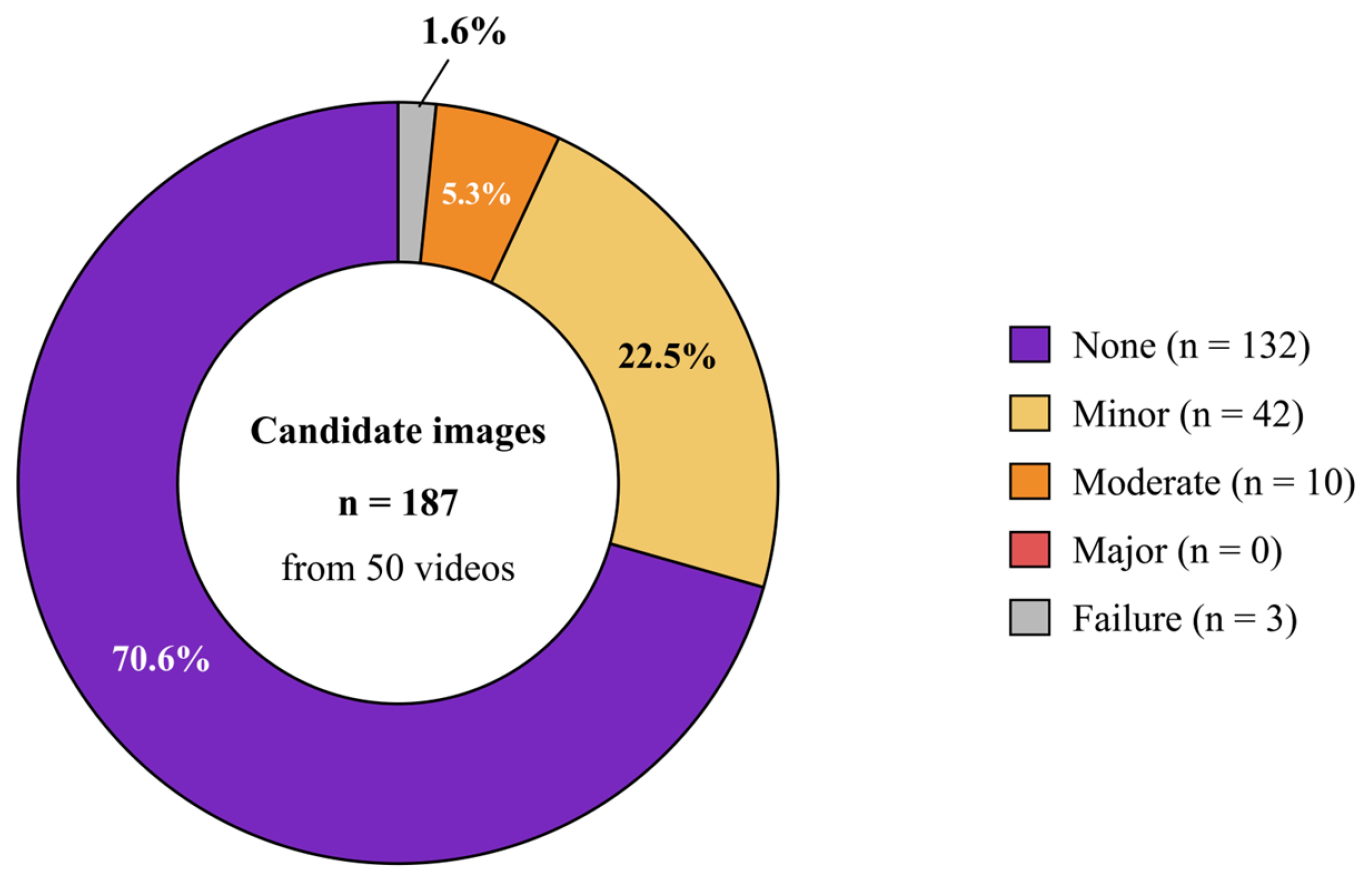
Distribution of expert-assessed agreement levels for video-based standard plane acquisition. None and Minor indicate clinically acceptable candidate images without discrepancies that affect standard plane determination or subsequent Graf angle measurement; Moderate and Major indicate progressively greater discrepancies that may compromise standard plane assessment or measurement reliability; Failure indicates that the captured candidate frame could not be accepted as a valid standard plane for subsequent analysis.

To evaluate the feasibility of applying the standard plane detection module in continuous scanning scenarios, 50 hip ultrasound videos from 50 unique infants were retrospectively selected. All videos were acquired during routine examinations by one experienced clinician, and each video included a continuous scanning sequence that passed through at least one Graf standard plane. The presence of a standard plane in each video was confirmed by the acquiring clinician and independently reviewed by two additional experienced clinicians. The trained YOLOv8-pose model was then applied to the video streams for frame-by-frame analysis. Whenever a frame satisfied the standard plane criteria, the system automatically saved it as a candidate standard plane image. A total of 187 candidate standard plane images were automatically captured. These candidate images were subsequently evaluated visually by clinical experts to determine whether they satisfied the Graf standard plane criteria. The distribution of the expert evaluation results is shown in Figure 7.

As shown in Figure 7, among the 187 candidate standard plane images, 132 were rated as None, accounting for 70.6%, and 42 were rated as Minor, accounting for 22.5%. Together, the None and Minor categories represented 93.1% of all candidate images, indicating that the vast majority showed no clinically relevant discrepancies from the expert assessments. In addition, 10 images were rated as Moderate (5.3%), no images were rated as Major, and 3 images were rated as Failure (1.6%).

At the video level, the system generated 3.74 ± 1.26 candidate frames per video, with a median of 4 and a range of 1–6 frames. Among the 50 videos, 48 contained at least one candidate frame rated as None or Minor by the clinical experts, corresponding to a video-level success rate of 96.0%. Together, the frame-level and video-level results support the feasibility of the proposed module for automatically identifying clinically acceptable candidate standard plane images during continuous ultrasound scanning and providing suitable input for subsequent key anatomical structure segmentation and Graf angle measurement.

**Figure 8:**
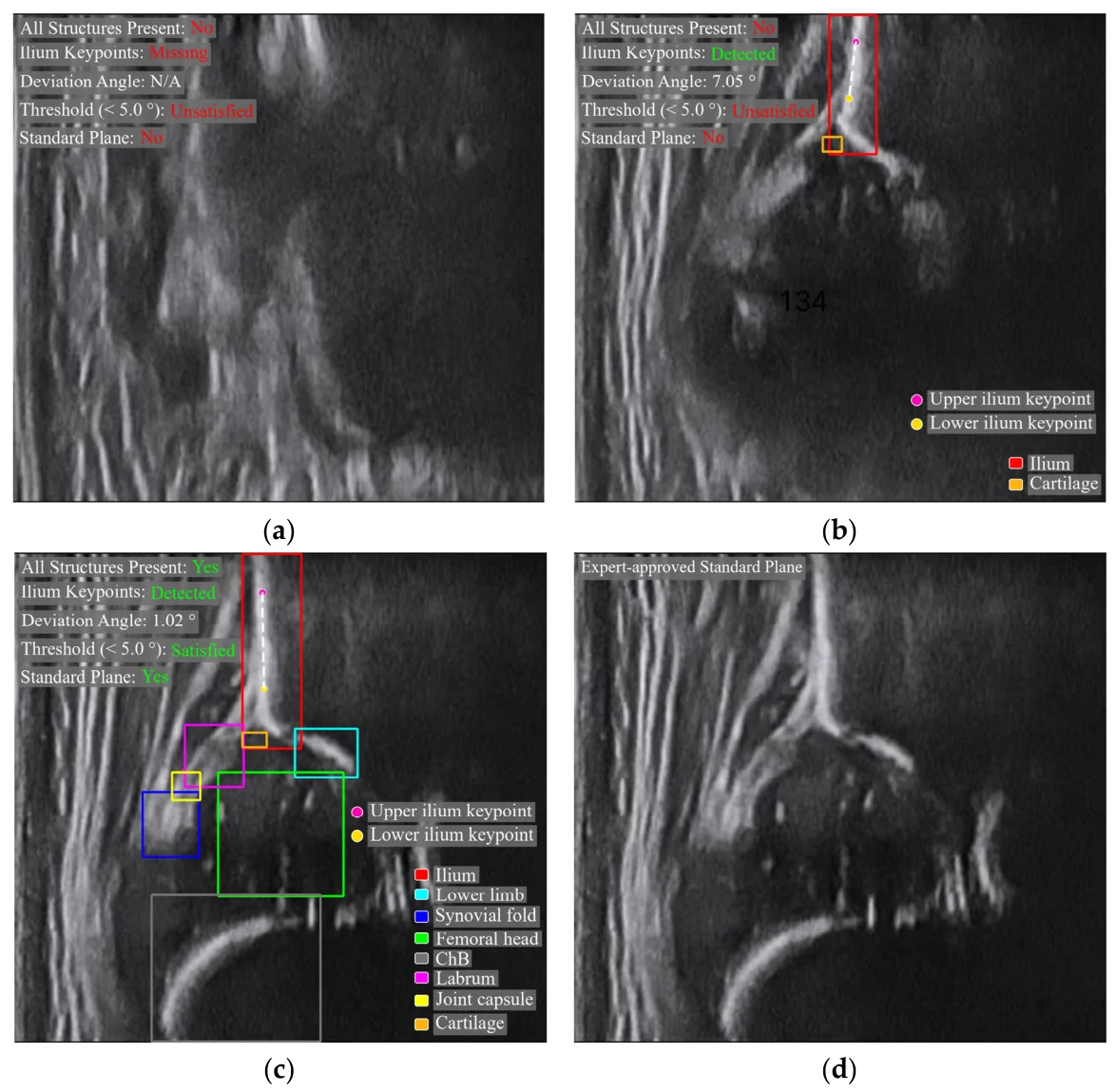
Representative example of video-based automatic standard plane acquisition. (**a**) Non-standard frame. (**b**) Near-standard frame. (**c**) Automatically saved candidate frame. (**d**) Expert-approved standard plane.

Figure 8 illustrates a representative process of automated standard plane acquisition from a continuous ultrasound video. Figure 8a shows a nonstandard frame that did not satisfy the standard plane criteria; Figure 8b shows a frame approaching the standard plane but not yet fully meeting the classification criteria; Figure 8c shows the candidate standard plane image automatically captured by the model; and Figure 8d shows the standard plane image confirmed by the expert. The model was able to identify the anatomical structures frame by frame during continuous scanning and automatically capture a candidate frame once the standard plane criteria were satisfied. Expert evaluation confirmed that the automatically captured candidate frame met the Graf standard plane requirements, demonstrating the practical feasibility of this module for assisting standard plane acquisition.

### 3.3. Comparison of Automated and Expert Measurements

**Figure 9:**
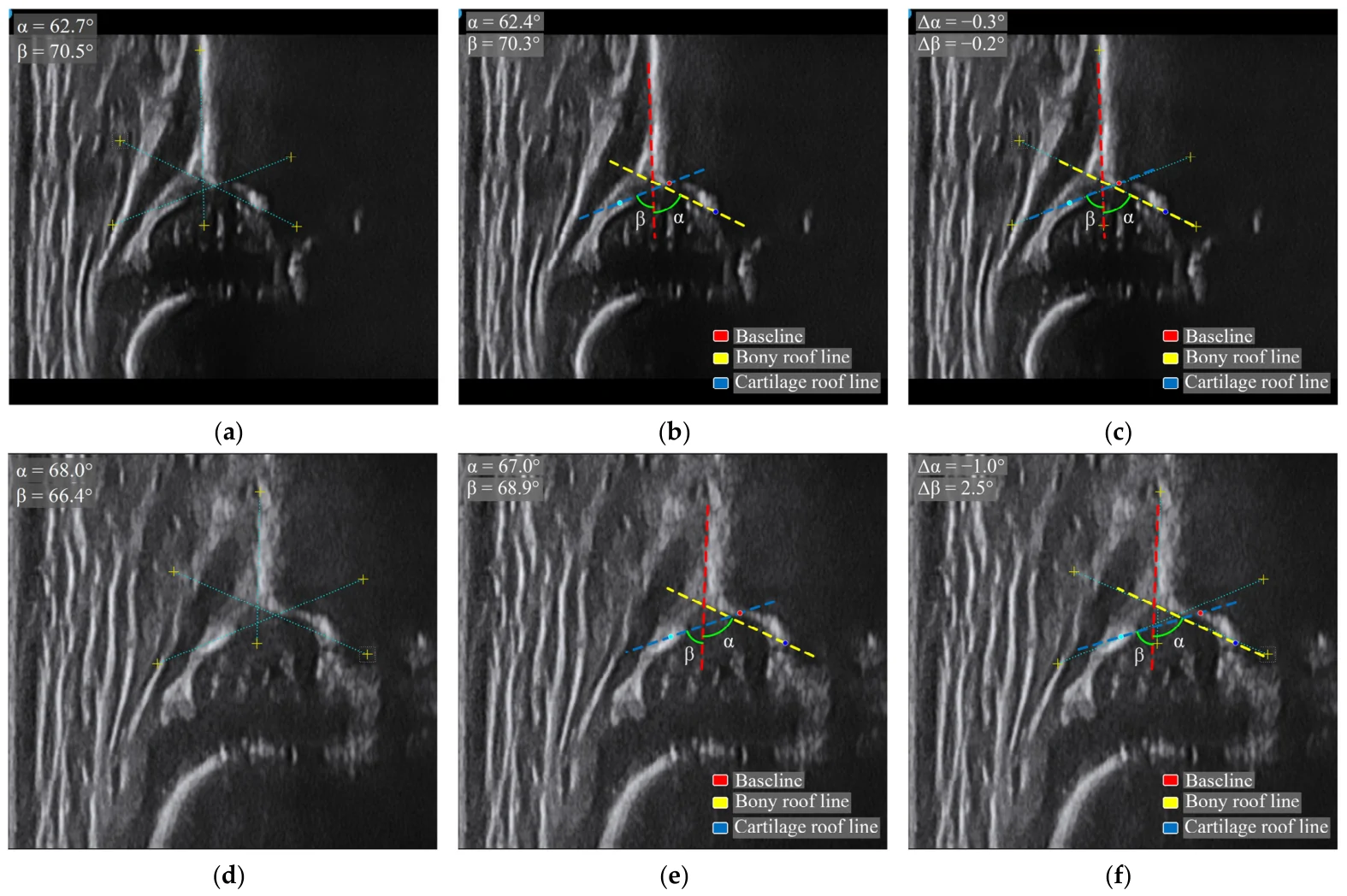
Representative comparisons of expert and automated Graf angle measurements. (**a**–**c**) A typical case with small measurement differences. (**d**–**f**) A challenging case with relatively large measurement differences. The columns show expert measurements, automated measurements, and their overlays, respectively. Cyan dashed lines indicate the expert-drawn reference lines, with yellow crosses marking the endpoints. Red, yellow, and blue dashed lines represent the baseline, bony roof line, and cartilage roof line, respectively. The differences between expert and automated measurements are displayed in the upper-left corner of the overlaid images. Δα and Δβ were calculated as the automated measurement minus the expert measurement.

The test set comprised 307 standard plane images, including 223 Graf type I images and 84 Graf type II images. The automated measurements were compared with the expert measurements, which served as the reference standard. As shown in Table 5, the automated and expert measurements were generally comparable. For the α angle, the mean absolute error (MAE) and root mean square error (RMSE) were 1.61° and 1.90°, respectively, whereas the corresponding values for the β angle were 2.13° and 2.43°, indicating that the measurement error was slightly greater for the β angle than for the α angle. Figure 9 presents the expert measurements, automated measurements, and their overlays for representative typical and challenging cases. Overall, the measurement lines generated by the automated method closely approximated those obtained by the expert.

**Figure 10:**
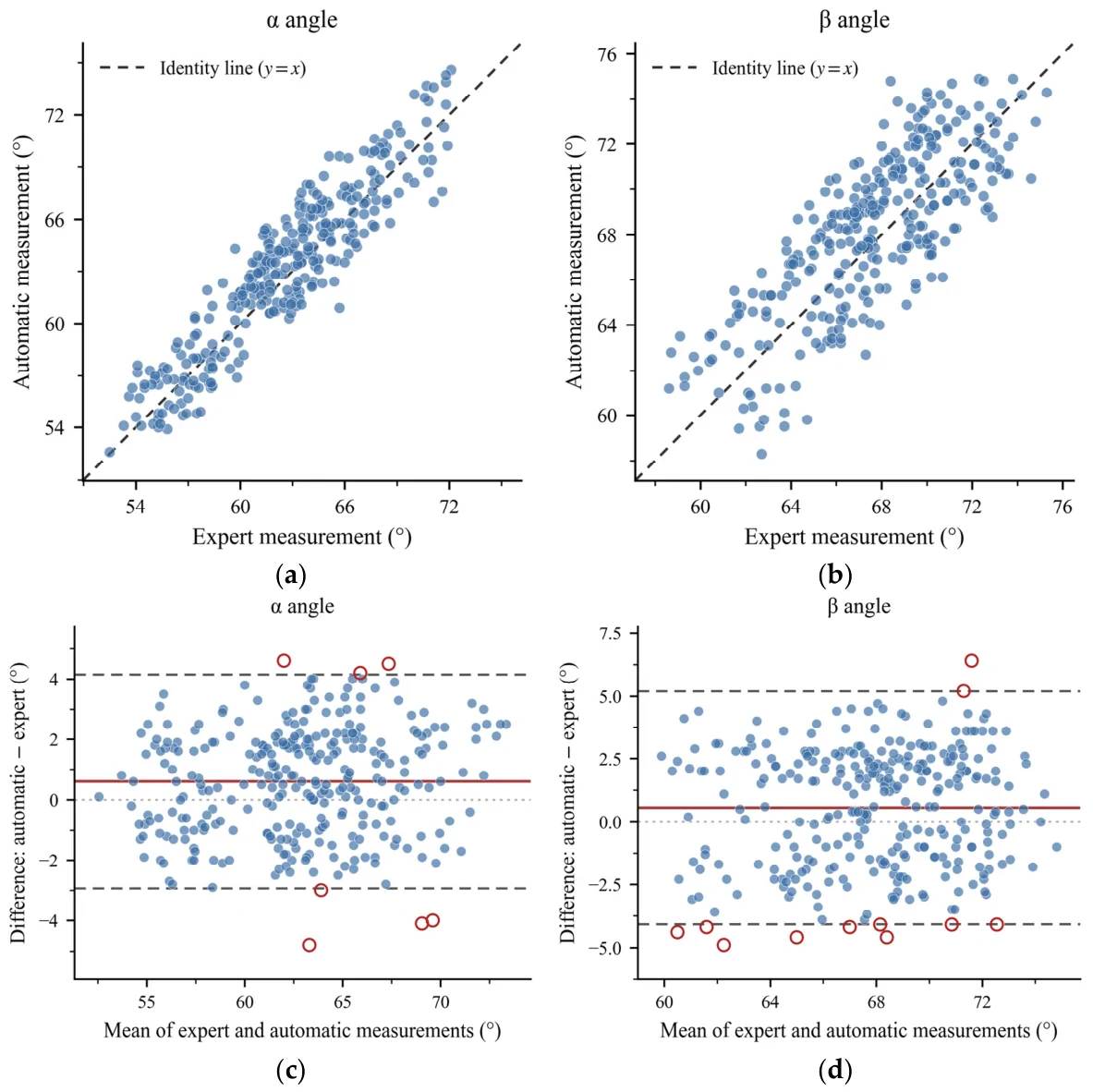
Agreement between expert and automated Graf angle measurements. (**a**) Scatter plot for the α angle. (**b**) Scatter plot for the β angle. The dashed diagonal lines represent the identity line y = x. (**c**) Bland–Altman plot for the α angle. (**d**) Bland–Altman plot for the β angle. The solid red lines indicate the mean bias, and the dashed gray lines indicate the 95% limits of agreement, and the red open circles indicate measurements outside the 95% limits of agreement.

Agreement between the two measurement methods was further evaluated using the intraclass correlation coefficient and Bland–Altman analysis, with the results summarized in Table 6. The mean biases for the α and β angles were 0.60° and 0.55°, respectively, indicating that the automated measurements were, on average, slightly higher than the expert measurements. The ICC for the α angle was 0.913, indicating excellent agreement, whereas that for the β angle was 0.766, indicating good agreement. The wider 95% limits of agreement observed for the β angle were consistent with its higher MAE and RMSE values. As shown in Figure 10, the data points for the α angle were more closely distributed around the line of identity, and the differences in the Bland–Altman plot were more tightly clustered. In contrast, the β-angle measurements exhibited greater dispersion and wider limits of agreement. Overall, the automated method demonstrated better agreement with expert measurements for the α angle than for the β angle.

Subgroup analysis was further performed according to the expert-derived Graf classification. As summarized in Table 7, for Graf type I hips, the MAEs of the α and β angles were 1.66° and 2.09°, respectively, with corresponding RMSE values of 1.96° and 2.38°. For Graf type II hips, the MAEs were 1.47° and 2.23°, with corresponding RMSE values of 1.75° and 2.54°. Overall, the measurement errors were comparable between Graf type I and II hips, indicating relatively stable automated measurement performance across the two Graf categories represented in the current dataset.

### 3.4. Computational Efficiency Analysis

Five ultrasound scanning videos and 200 ultrasound images were randomly selected from the test set for computational efficiency evaluation. All experiments were conducted on a computing platform equipped with an AMD Ryzen 7 5800H CPU, 16 GB of RAM, and an NVIDIA GeForce RTX 3060 Laptop GPU with 6 GB of video memory. The software environment consisted of Windows 10, Python 3.8, and PyTorch 2.3.1. Based on this experimental platform, the real-time performance of the standard plane detection model, the computational efficiency of ROI-based and full-image segmentation, and the end-to-end runtime efficiency of the complete DDH-Net pipeline were evaluated separately.

#### 3.4.1. Real-Time Performance of Standard Plane Detection

Each of the five test videos was processed frame by frame and independently evaluated three times under identical experimental conditions. The mean of the three repeated measurements was taken as the final result for each video. The mean per-frame processing time and processing speed were then calculated across the five videos. As shown in Table 8, the original frame rates of the test videos ranged from 32 to 33 FPS. The mean per-frame processing time across the individual videos ranged from 14.16 to 15.08 ms, corresponding to processing speeds of 66.34–70.66 FPS. Overall, the standard plane detection module achieved a mean per-frame processing time of 14.61 ± 0.34 ms and a mean processing speed of 68.51 ± 1.57 FPS. The relatively small variations in processing time and speed across the videos indicate stable runtime performance during continuous ultrasound scanning. Moreover, the processing speed exceeded the original frame rates of the test videos, demonstrating the ability of the detection module to support real-time, frame-by-frame standard plane detection and automatic capture of candidate images. The end-to-end computational efficiency of the complete DDH-Net pipeline is evaluated separately in Section 3.4.3.

#### 3.4.2. Efficiency Comparison Between ROI-Based and Full-Image Segmentation

**Figure 11:**
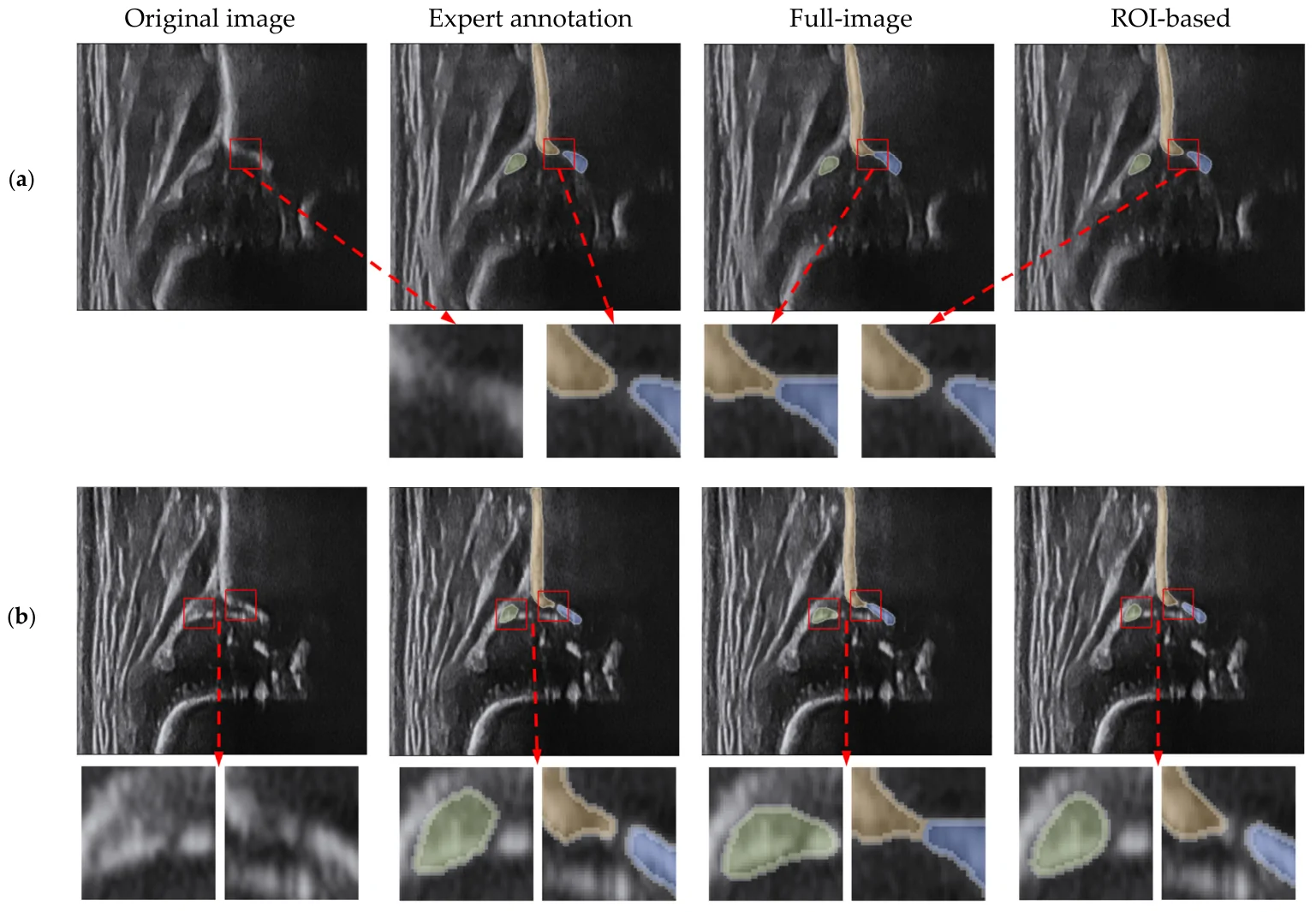
Comparison between full-image segmentation and ROI-based segmentation. (**a**) Representative example 1; (**b**) Representative example 2. Original ultrasound images, expert annotations, full-image segmentation results, and ROI-based segmentation results are shown from left to right. The enlarged regions highlight differences in the delineation of small anatomical structures. The ilium, labrum, and lower limb of the ilium are indicated using consistent colors across all panels.

The 200 test images described above were used to compare the performance of full-image segmentation with that of the ROI-based segmentation strategy. The two approaches used the same U-Net architecture, dataset split, training hyperparameters, loss function, and evaluation metrics, differing only in the input strategy and corresponding image preprocessing. Both methods were independently evaluated three times under identical experimental conditions, and the segmentation accuracy and mean processing time per image were recorded. As shown in Table 9, the ROI-based method achieved higher DSC values and lower HD values for the ilium, labrum, and lower limb of the ilium than the full-image segmentation method, indicating improved region overlap and boundary localization accuracy. Meanwhile, the mean processing time per image decreased from 24.78 ± 1.57 ms to 16.44 ± 0.94 ms, representing a reduction of 33.7%. The processing speed increased by approximately 50.7%, reaching approximately 1.51 times that of full-image segmentation. Figure 11 further presents representative segmentation results obtained using the two methods. Although both methods successfully identified the major anatomical structures, the ROI-based method reduced false segmentation in background regions in some images and produced clearer structural boundaries.

#### 3.4.3. End-to-End Efficiency and System-Level Lightweight Evaluation

The proposed DDH-Net comprises four stages: standard plane detection based on object detection and pose estimation, ROI cropping and preprocessing, key anatomical structure segmentation, and Graf angle measurement. Table 10 and Table 11 evaluate DDH-Net in terms of computational resource consumption and runtime efficiency, respectively. The standard plane detection model and ROI segmentation model contained 3.08 million and 43.93 million parameters, respectively, resulting in a total of 47.01 million parameters for the complete DDH-Net. Under FP32 precision with a batch size of 1, the peak GPU memory usage was 254.14 MB. Most of the parameters and GPU memory consumption were attributable to the segmentation module. Nevertheless, the peak memory usage of the end-to-end pipeline was only slightly higher than that observed when the segmentation task was executed independently, indicating that sequential execution of the individual stages enabled effective reuse of computational resources.

The complete DDH-Net pipeline achieved a mean processing time of 34.64 ± 1.10 ms per image, corresponding to an end-to-end processing speed of approximately 28.87 FPS. Standard plane detection and key anatomical structure segmentation accounted for 43.4% and 45.4% of the total processing time, respectively, and were therefore the primary computational bottlenecks. In contrast, ROI extraction and preprocessing accounted for only 2.0% of the total processing time and introduced negligible additional computational overhead. These results demonstrate that DDH-Net provides near-real-time ultrasound image analysis with limited GPU memory consumption and exhibits favorable overall computational efficiency.

## 4. Discussion

This study proposed DDH-Net, a fully automated computer-aided assessment method for infant hip ultrasonography that follows the Graf diagnostic workflow and sequentially performs standard plane detection, ROI extraction, key anatomical structure segmentation, and Graf angle measurement. The results demonstrated that the proposed method could reliably exclude non-standard planes, automatically identify candidate images with high clinical acceptability from continuous ultrasound scanning videos, and achieve α- and β-angle measurements that were generally consistent with expert measurements. In addition, the ROI-driven segmentation strategy improved the segmentation performance of small-scale anatomical structures while reducing the computational cost of the segmentation stage.

Reliable identification of the standard plane is a prerequisite for accurate Graf angle measurement. DDH-Net not only evaluates whether all eight anatomical structures are fully visualized but also calculates the ilium inclination angle based on two ilium orientation keypoints, thereby assessing the degree of imaging plane tilt. The standard plane detection module reliably distinguished standard from nonstandard planes, with no false-positive predictions observed in the test set. In clinical practice, a missed standard plane can generally be compensated for by continued scanning or selection of an alternative candidate frame, whereas false acceptance of a nonstandard plane may directly compromise subsequent Graf angle measurement. Chen et al. [23] evaluated standard plane quality using local anatomical structures, including the ilium, labrum, lower limb of the ilium, and bony rim, and reported a classification accuracy of 94.71%. Compared with their local structure-based assessment method, the present study further incorporates both the completeness of anatomical structures and constraints on ilium orientation, more closely reflecting the definition of a standard plane in the Graf method. The small number of false-negative results may have arisen from incomplete visualization of local structures or errors in ilium keypoint localization. Such errors generally prevent the affected frame from entering the subsequent measurement pipeline rather than directly producing an erroneous Graf angle measurement.

The video-based evaluation further showed that clinically acceptable candidate standard planes could be identified in most videos, supporting the feasibility of extending the standard plane detection module from preselected static images to continuous ultrasound scanning. The unsuccessful cases were mainly associated with a short duration of standard plane appearance during scanning and relatively poor image quality, which reduced the likelihood of capturing candidate frames that fully satisfied the predefined criteria. This functionality may reduce the operational burden associated with repeatedly pausing the examination, selecting frames, and saving images, while potentially providing guidance on standard plane acquisition for less-experienced examiners. However, the video experiments in this study were limited to offline frame-by-frame processing and visual assessment of the candidate images. Whether the proposed module can shorten the actual examination time, improve the operator learning curve, or increase the success rate of the initial examination remains to be validated in prospective clinical studies.

The ROI-driven segmentation strategy improved segmentation performance for the ilium, labrum, and lower limb of the ilium while reducing computational cost compared with full-image segmentation. This improvement may be attributed to the local cropping procedure, which reduces background interference from the femoral head, muscles, and other irrelevant tissues while increasing the relative proportion of small-scale structures, such as the labrum and lower limb of the ilium, within the input image. However, ROI-based segmentation depends on the accuracy of the preceding anatomical structure detection. Although no ROI-exclusion failures were observed in the current test set, the 10% margin expansion and training with detection-derived ROIs were adopted to reduce sensitivity to moderate localization errors. Nevertheless, substantial detection errors could still exclude clinically relevant structures and adversely affect subsequent segmentation and Graf angle measurement. This potential source of error propagation should be further evaluated in larger and more diverse datasets.

The automated measurements were generally comparable to the expert measurements, although agreement was higher for the α angle than for the β angle. In this study, the MAEs for the α and β angles were 1.61° and 2.13°, respectively, while the corresponding ICC values were 0.913 and 0.766. Measurement of the α angle primarily depends on the iliac contour and the lower limb of the ilium, which are osseous structures that typically exhibit relatively clear and continuous hyperechoic boundaries. In contrast, measurement of the β angle relies on localization of the labrum and the bony rim point. In challenging anatomical cases, the labrum may be small, poorly defined, partially obscured, or affected by speckle noise and local loss of echogenicity, making its segmentation and center localization less stable. In addition, morphological variability of the labrum and bony rim may further increase localization uncertainty. Because the cartilage roof line is constructed from the bony rim point to the labral center, even a small displacement of either landmark can alter its orientation and consequently produce a larger variation in the β angle.

The subgroup analysis further showed generally comparable measurement errors between Graf type I and type II hips. The α-angle measurement errors were slightly lower in Graf type II hips, whereas the β-angle errors were slightly higher; however, the overall differences between the two subgroups were small. These findings suggest relatively stable automated angle measurement performance across the Graf type I and II cases represented in the current dataset. However, because no Graf type III or IV hips were available, the performance of DDH-Net in more severe DDH cases remains to be established. From a clinical perspective, although the overall angle measurement errors were relatively small, their clinical impact may be greater in cases close to Graf classification boundaries, where small angular deviations could potentially alter the assigned Graf type. The present study primarily focused on the accuracy and agreement of automated angle measurements rather than on classification performance. Therefore, a dedicated evaluation of Graf classification agreement, particularly for borderline cases and across a broader range of DDH severity, will therefore be an important direction for future work.

Compared with previously reported automated methods summarized in Table 12, DDH-Net showed relatively low measurement errors and comparable agreement with expert measurements, with better agreement observed for the α angle than for the β angle. However, direct cross-study comparisons should be interpreted cautiously because of differences in datasets, imaging systems, and evaluation protocols. The lack of a common public benchmark and standardized implementations also limits direct head-to-head comparison with existing methods. In addition, because the clinical experts in this study contributed to establishing the reference measurements, the current design does not provide an independent clinician-versus-DDH-Net comparison. Therefore, future prospective reader studies and standardized same-dataset evaluations are needed to establish relative clinical advantage. The results for the β angle further suggest that labrum segmentation and cartilaginous roof line construction remain major sources of error in current methods. Future work may improve the stability of automated β-angle measurement by increasing the number of challenging labral cases, introducing boundary-constrained loss functions, optimizing the localization of the labral center, and incorporating indications of measurement uncertainty.

Another distinctive feature of DDH-Net is its integration of standard plane quality control, key anatomical structure segmentation, and interpretable geometric measurement within a unified workflow. The method first screens images that satisfy the standard plane criteria and extracts the contours of the key anatomical structures. It then localizes the measurement landmarks according to the Graf method and automatically constructs the baseline, bony roof line, and cartilage roof line. Clinicians can directly inspect the segmentation results, measurement landmarks, and reference lines, thereby assessing the reliability of the automated measurements and tracing potential sources of error. Manual expert review may be warranted when anatomical segmentation or Graf reference-line construction appears unreliable, or when the measured angles are close to classification boundaries; quantitative rejection thresholds remain to be established in future studies. In addition, the complete pipeline maintained near-real-time processing with manageable GPU memory consumption, supporting the computational feasibility of the proposed multistage framework.

This study has several limitations. First, it was a single-center retrospective study, and all images were acquired using the same ultrasound system. Ultrasound appearance may vary across imaging devices, acquisition settings, and operators. In addition, the current dataset was not designed or sufficiently diverse to support systematic subgroup analyses of potential performance variations associated with patient age, sex, hip laterality, or acquisition-related factors. Therefore, the robustness and generalizability of DDH-Net across different patient populations and clinical environments remain to be established through larger multicenter, cross-device, and adequately stratified validation studies. Second, the standard plane dataset included only Graf type I and II hips, with no Graf type III or IV cases available for evaluation. Therefore, the present findings establish the performance of DDH-Net only within the range of Graf type I and II cases included in this study, whereas its performance in severe dysplasia, dislocated hips, and other complex cases remains to be established. Third, the video experiments primarily evaluated the clinical acceptability of automatically saved candidate frames, whereas the performance of the method within a prospective clinical workflow has not yet been investigated. Fourth, agreement for the β angle remained lower than that for the α angle, indicating that the segmentation of small-scale soft-tissue boundaries and localization of the labral center require further improvement. Future studies should include larger multicenter, cross-device, and adequately stratified datasets to systematically evaluate robustness across patient characteristics and acquisition conditions, increase the number of Graf type III and IV cases, and incorporate uncertainty alerts and expert review mechanisms to further improve the clinical reliability of the proposed method.

## 5. Conclusions

This study proposed DDH-Net, a fully automated computer-aided assessment method for infant hip ultrasonography that follows the Graf workflow and integrates standard plane detection, ROI extraction, key anatomical structure segmentation, and automated measurement of the α and β angles. The results demonstrated that DDH-Net could effectively exclude non-standard planes and automatically acquire candidate images with high clinical acceptability during continuous ultrasound scanning. The ROI-driven segmentation strategy improved the segmentation performance of key anatomical structures while reducing the computational cost of the segmentation stage. Automated α-angle measurements showed good agreement with expert measurements, whereas the lower agreement observed for the β angle indicates that labrum segmentation and bony rim localization require further improvement. DDH-Net follows the Graf workflow, sequentially performing standard plane determination, key anatomical structure segmentation, measurement landmark localization, and reference line construction. Intermediate results from each stage are visualized, thereby enhancing the interpretability and verifiability of the automated analysis process. The present findings are limited to Graf type I and II hips, and the performance of DDH-Net in Graf type III and IV hips or dislocated hips remains to be established. Moreover, the system achieves near-real-time processing under limited GPU resources. Collectively, these findings demonstrate the technical feasibility and measurement performance of DDH-Net within the population evaluated in this study. From a clinical perspective, DDH-Net may have potential to support efficient and consistent infant hip ultrasound assessment; however, its generalizability and actual clinical benefits remain to be established through multicenter, cross-device, and prospective clinical studies. Future work will expand the sample size of Graf type III and IV hips and other complex cases and further optimize labrum segmentation and β-angle measurement to improve the applicability and clinical reliability of DDH-Net across different DDH types.

## Figures and Tables

**Table 1:** Dataset allocation for standard plane detection, segmentation of key anatomical structures, Graf angle measurement, and video-based evaluation.

Type	Training Set	Validation Set	Test Set	Total
Infants	984	143	282	1409
Total images	1854	265	529	2648
Standard plane images	1072	153	307	1532
Nonstandard plane images	782	112	222	1116
Images for YOLOv8-pose	1854	265	529	2648
Images for U-Net	1072	153	307	1532
Images for Graf angle measurement	—	—	307	307
Ultrasound videos	—	—	50	50

Total images include both standard plane and non-standard plane ultrasound images. YOLOv8-pose was trained and evaluated using the complete image dataset, whereas U-Net was trained and evaluated only on the standard plane subset. Graf angle measurement was evaluated on the standard plane images in the test set. The 50 ultrasound videos were not used for model training and were only used for qualitative evaluation of real-time performance. Dataset partitioning was performed at the infant level, and all images from the same infant were retained within the same subset.

**Table 2:** Distribution of Graf types in standard plane ultrasound images.

Graf Type	Training Set	Validation Set	Test Set	Total
Graf type I	779	111	223	1113
Graf type II	293	42	84	419
Total	1072	153	307	1532

Graf type distribution was calculated only for standard plane ultrasound images and was determined according to expert manual measurements of the α and β angles.

**Table 3:** Confusion matrix of standard plane detection.

Expert Reference Standard	Predicted Standard Plane	Predicted Nonstandard Plane	Total
Standard plane	290	17	307
Nonstandard plane	0	222	222
Total	290	239	529

Standard plane was defined as the positive class.

**Table 4:** Performance metrics for standard plane detection.

Metric	Value
Accuracy	96.8%
Sensitivity	94.5%
Specificity	100.0%
Positive predictive value	100.0%
Negative predictive value	92.9%
F1-score	97.2%
Cohen’s kappa	0.935

**Table 5:** Comparison of expert and automated Graf angle measurements.

Angle	Expert Measurement (°)	Automated Measurement (°)	MAE (°)	RMSE (°)
α	62.53 ± 4.43	63.13 ± 4.67	1.61	1.90
β	67.62 ± 3.45	68.17 ± 3.60	2.13	2.43

Data are presented as mean ± standard deviation. The sample size was 307 for each angle. MAE, mean absolute error; RMSE, root mean square error.

**Table 6:** Agreement between expert and automated Graf angle measurements.

Angle	Bias (°)	95% LoA (°)	ICC (95% CI)	Agreement
α	0.60	−2.94 ~ 4.15	0.913 (0.880–0.940)	Excellent
β	0.55	−4.09 ~ 5.19	0.766 (0.710–0.810)	Good

Bias was calculated as automated measurement minus expert measurement. ICC was calculated using a two-way mixed-effects model with absolute agreement and single measurements [ICC(A,1)]. LoA, limits of agreement; CI, confidence interval.

**Table 7:** Subgroup analysis of automated Graf angle measurements according to Graf classification.

Graf Type	n	α MAE (°)	α RMSE (°)	β MAE (°)	β RMSE (°)
Graf I	223	1.66	1.96	2.09	2.38
Graf II	84	1.47	1.75	2.23	2.54

**Table 8:** Real-time performance of standard plane detection across five ultrasound videos.

Video	Frames	Processing Time per Frame (ms)	Processing Speed (FPS)
Video 1	890	14.16 ± 0.43	70.66 ± 2.18
Video 2	857	14.74 ± 0.61	67.91 ± 2.76
Video 3	1048	14.51 ± 0.47	68.97 ± 2.19
Video 4	930	14.57 ± 0.40	68.65 ± 1.86
Video 5	1041	15.08 ± 0.41	66.34 ± 1.80
Overall	4766	14.61 ± 0.34	68.51 ± 1.57

**Table 9:** Comparison of segmentation performance and computational efficiency between full-image and ROI-based segmentation.

Method	Anatomical Structure	DSC	HD (Pixels)	Time per Image (ms)
Full-image	Ilium	0.8917	7.1436	24.78 ± 1.57
	Labrum	0.8280	4.4907	
	Lower limb	0.8324	6.1238	
ROI-based	Ilium	0.9028	6.4342	16.44 ± 0.94
	Labrum	0.8699	3.7233	
	Lower limb	0.8763	4.5087	

DSC, Dice similarity coefficient; HD, Hausdorff distance. DSC and HD were averaged across 200 test images. Processing time is presented as the mean ± standard deviation of three repeated runs. For ROI-based segmentation, the processing time included ROI cropping, image preprocessing, U-Net inference, removal of letterbox padding, and restoration of the predicted mask to the original image coordinates; For full-image segmentation, the processing time included image preprocessing, U-Net inference, and restoration of the predicted mask to the original image size.

**Table 10:** Model complexity and resource requirements of DDH-Net.

Component	Input Size	Parameters (M)	Peak GPU Memory (MB)
Standard plane detection	416 × 416	3.08	50.85
ROI-based segmentation	192 × 256	43.93	239.39
Complete pipeline	416 × 416/192 × 256	47.01	254.14

**Table 11:** End-to-end computational efficiency of the proposed method.

Stage	Time (ms), Mean ± SD	Proportion (%)
Detection	15.03 ± 0.31	43.4
ROI + preprocessing	0.71 ± 0.04	2.0
Segmentation	15.73 ± 0.93	45.4
Graf angle measurement	3.17 ± 0.45	9.2
End-to-end pipeline	34.64 ± 1.10	100.0

**Table 12:** Comparison of automated Graf angle measurement methods reported in previous studies.

Study	Dataset Size	α-Angle MAE (°)	β-Angle MAE (°)	α-Angle ICC	β-Angle ICC
Hu et al. [18]	247	2.22	2.89	—	—
Lee et al. [19]	320	—	—	0.764	0.743
Huang et al. [20]	316	2.30	2.49	0.790	0.870
Chen et al. [23]	1051	1.71	2.40	0.850	0.730
DDH-Net	307	1.61	2.13	0.913	0.766

## Data Availability

The data supporting the findings of this study are available from the corresponding author upon reasonable request, subject to approval by the relevant ethics committee and data-owning institution. Owing to privacy, ethical, and institutional restrictions associated with clinical ultrasound examinations involving infants, the raw data are not publicly available.

## Abbreviations

The following abbreviations are used in this manuscript:

DDH	developmental dysplasia of the hip
ROI	Region of interest
MAE	Mean absolute error
ICC	Intraclass correlation coefficient
CNNs	Convolutional neural networks
ChB	Chondro-osseous border
RMSE	Root mean square error
LoA	Limits of agreement
CI	Confidence interval
DSC	Dice similarity coefficient
HD	Hausdorff distance

## References

[1] Weinstein S.L., Mubarak S.J., Wenger D.R. (2003). Developmental hip dysplasia and dislocation: Part I. J. Bone Jt. Surg..

[2] Loder R.T., Skopelja E.N. (2011). The epidemiology and demographics of hip dysplasia. Int. Sch. Res. Not..

[3] Engesæter I.Ø., Lie S.A., Lehmann T.G., Furnes O., Vollset S.E., Engesæter L.B. (2008). Neonatal hip instability and risk of total hip replacement in young adulthood. Acta Orthop..

[4] Barrera C.A., Cohen S.A., Sankar W.N., Ho-Fung V.M., Sze R.W., Nguyen J.C. (2019). Imaging of developmental dysplasia of the hip: Ultrasound, radiography and magnetic resonance imaging. Pediatr. Radiol..

[5] Graf R. (1980). The diagnosis of congenital hip-joint dislocation by the ultrasonic Combound treatment. Arch. Orthop. Trauma. Surg..

[6] Graf R. (2006). Hip Sonography: Diagnosis and Management of Infant Hip Dysplasia.

[7] Graf R., Lercher K., Scott S., Spieß T. (2017). Essentials of Infant Hip Sonography According to Graf.

[8] Dias J.J., Thomas I.H., Lamont A.C., Mody B.S., Thompson J.R. (1993). The reliability of ultrasonographic assessment of neonatal hips. J. Bone Jt. Surg. Br..

[9] Roovers E.A., Boere-Boonekamp M.M., Geertsma T.S.A., Zielhuis G.A., Kerkhoff A.H.M. (2003). Ultrasonographic screening for developmental dysplasia of the hip in infants: Reproducibility of assessments made by radiographers. J. Bone Jt. Surg. Br..

[10] Quader N., Hodgson A.J., Mulpuri K., Savage T., Abugharbieh R. (2015). Automatic assessment of developmental dysplasia of the hip. Proceedings of the 2015 IEEE 12th International Symposium on Biomedical Imaging (ISBI 2015), Brooklyn, NY, USA, 16–19 April 2015.

[11] Quader N., Hodgson A.J., Mulpuri K., Schaeffer E., Abugharbieh R. (2017). Automatic evaluation of scan adequacy and dysplasia metrics in 2-D ultrasound images of the neonatal hip. Ultrasound Med. Biol..

[12] Hareendranathan A.R., Mabee M., Punithakumar K., Noga M., Jaremko J.L. (2016). Toward automated classification of acetabular shape in ultrasound for diagnosis of DDH: Contour alpha angle and the rounding index. Comput. Methods Programs Biomed..

[13] Sezer H.B., Sezer A. (2019). Automatic segmentation and classification of neonatal hips according to Graf’s sonographic method: A computer-aided diagnosis system. Appl. Soft Comput..

[14] Zhang Z., Tang M., Cobzas D., Zonoobi D., Jagersand M., Jaremko J.L. (2018). End-to-end detection-segmentation network with ROI convolution. Proceedings of the 2018 IEEE 15th International Symposium on Biomedical Imaging (ISBI 2018), Washington, DC, USA, 4–7 April 2018.

[15] El-Hariri H., Mulpuri K., Hodgson A., Garbi R. (2019). Comparative evaluation of hand-engineered and deep-learned features for neonatal hip bone segmentation in ultrasound. Medical Image Computing and Computer Assisted Intervention—MICCAI 2019.

[16] Pulik Ł., Czech P., Kaliszewska J., Mulewicz B., Pykosz M., Wiszniewska J., Łęgosz P. (2025). Artificial intelligence algorithm supporting the diagnosis of developmental dysplasia of the hip: Automated ultrasound image segmentation. J. Clin. Med..

[17] Golan D., Donner Y., Mansi C., Jaremko J., Ramachandran M., on behalf of CUDL (2016). Fully automating Graf’s method for DDH diagnosis using deep convolutional neural networks. Deep Learning and Data Labeling for Medical Applications.

[18] Hu X., Wang L., Yang X., Zhou X., Xue W., Cao Y., Liu S., Huang Y., Guo S., Shang N. (2022). Joint landmark and structure learning for automatic evaluation of developmental dysplasia of the hip. IEEE J. Biomed. Health Inform..

[19] Lee S.-W., Ye H.-U., Lee K.-J., Jang W.-Y., Lee J.-H., Hwang S.-M., Heo Y.-R. (2021). Accuracy of new deep learning model-based segmentation and key-point multi-detection method for ultrasonographic developmental dysplasia of the hip (DDH) screening. Diagnostics.

[20] Huang B., Xia B., Qian J., Zhou X., Zhou X., Liu S., Chang A., Yan Z., Tang Z., Xu N. (2023). Artificial intelligence-assisted ultrasound diagnosis on infant developmental dysplasia of the hip under constrained computational resources. J. Ultrasound Med..

[21] Liu R., Liu M., Sheng B., Li H., Li P., Song H., Zhang P., Jiang L., Shen D. (2021). NHBS-Net: A feature fusion attention network for ultrasound neonatal hip bone segmentation. IEEE Trans. Med. Imaging.

[22] Ahn K.-S., Choi J.H., Kwon H., Lee S., Cho Y., Jang W.Y. (2025). Deep learning-based automated guide for defining a standard imaging plane for developmental dysplasia of the hip screening using ultrasonography: A retrospective imaging analysis. BMC Med. Inform. Decis. Mak..

[23] Chen T., Zhang Y., Wang B., Wang J., Cui L., He J., Cong L. (2022). Development of a fully automated Graf standard plane and angle evaluation method for infant hip ultrasound scans. Diagnostics.

[24] Sioutis S., Kolovos S., Papakonstantinou M.-E., Altsitzioglou P., Polyzou M., Chlapoutakis K., Karampikas V., Gavriil P., Mitsiokapa E., Koulalis D. (2025). Impact of probe tilt on Graf ultrasonography accuracy for neonatal hip dysplasia screening. SICOT-J.

[25] Terven J., Córdova-Esparza D.-M., Romero-González J.-A. (2023). A comprehensive review of YOLO architectures in computer vision: From YOLOv1 to YOLOv8 and YOLO-NAS. Mach. Learn. Knowl. Extr..

[26] Ronneberger O., Fischer P., Brox T. (2015). U-Net: Convolutional networks for biomedical image segmentation. Medical Image Computing and Computer-Assisted Intervention—MICCAI 2015.

[27] Galea R.-R., Diosan L., Andreica A., Popa L., Manole S., Bálint Z. (2021). Region-of-interest-based cardiac image segmentation with deep learning. Appl. Sci..

[28] Li S., Liu J., Song Z. (2022). Brain tumor segmentation based on region of interest-aided localization and segmentation U-Net. Int. J. Mach. Learn. Cybern..

[29] Xie J., Zhang S., Lu J., Luo Y. (2021). L-SNet: From region localization to scale-invariant medical image segmentation. Proceedings of the 2021 IEEE International Conference on Image Processing (ICIP), Anchorage, AK, USA, 19–22 September 2021.

[30] Koo T.K., Li M.Y. (2016). A guideline of selecting and reporting intraclass correlation coefficients for reliability research. J. Chiropr. Med..

[31] Gerke O. (2020). Reporting standards for a Bland–Altman agreement analysis: A review of methodological reviews. Diagnostics.

